# Methylene Blue Dye as Photosensitizer for Scavenger-Less Water Photo Splitting: New Insight in Green Hydrogen Technology

**DOI:** 10.3390/polym14030523

**Published:** 2022-01-27

**Authors:** Nasser A. M. Barakat, Gehan M. K. Tolba, Khalil Abdelrazek Khalil

**Affiliations:** 1 Chemical Engineering Department, Faculty of Engineering, Minia University, Minia 61519, Egypt; jehan.kotb@mu.edu.eg; 2 Department of Mechanical & Nuclear Engineering, College of Engineering, University of Sharjah, Sharjah 27272, United Arab Emirates; kabdelmawgoud@sharjah.ac.ae

**Keywords:** photosensitizer, hydrogen, water photo splitting, electrospinning, silica nanoparticles

## Abstract

In this study, hydrogen generation was performed by utilizing methylene blue dye as visible-light photosensitizer while the used catalyst is working as a transfer bridge for the electrons to H^+^/H_2_ reaction. Silica NPs-incorporated TiO_2_ nanofibers, which have a more significant band gap and longer electrons lifetime compared to pristine TiO_2_, were used as a catalyst. The nanofibers were prepared by electrospinning of amorphous SiO_2_ NPs/titanium isopropoxide/poly (vinyl acetate)/N, N-dimethylformamide colloid. Physicochemical characterizations confirmed the preparation of well morphology SiO_2_–TiO_2_ nanofibers with a bandgap energy of 3.265 eV. Under visible light radiation, hydrogen and oxygen were obtained in good stoichiometric rates (9.5 and 4.7 mL/min/gcat, respectively) without any considerable change in the dye concentration, which proves the successful exploitation of the dye as a photosensitizer. Under UV irradiation, SiO_2_ NPs incorporation distinctly enhanced the dye photodegradation, as around 91 and 94% removal efficiency were obtained from TiO_2_ nanofibers containing 4 and 6 wt% of the used dopant, respectively, within 60 min.

## 1. Introduction

One of the most critical processes in the developing renewable energy industry is converting of solar energy to chemical energy. Water splitting over particulate semiconductor catalysts has been touted as a cost-effective and straightforward approach for large-scale hydrogen generation. Water photo splitting is an electron transfer process between a donor (OH^−^ or H_2_O) and an acceptor (H^+^ or H_2_O) as follows:

*Donor    * 2H_2_O → O_2_ + 4H^+^ + 4e^−^   pH = 0    *E*_0_ = 1.229 V (1)

4OH^−^ → O_2_ + 2H_2_O + 4e^−^   pH = 14    *E*_0_ = 0.401 V (2)

* Acceptor    * 2H^+^ + 2e^−^ → H_2_     pH = 0    *E*_0_ = 0 V (3)

2H_2_O + 2e^−^ → H_2_ + OH^−^     pH = 14    *E*_0_ = −0.829 V (4)

**Figure 1:**
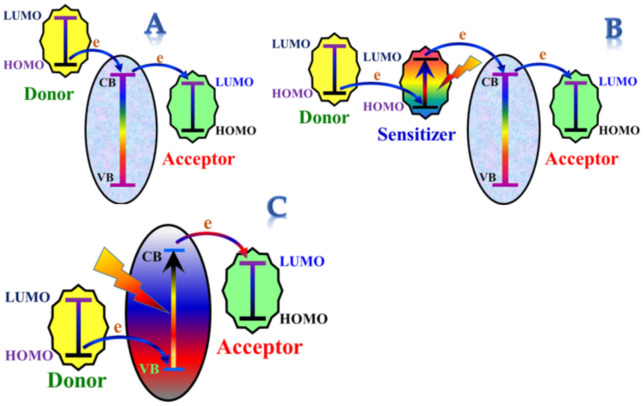
Conceptual illustration for the electron transfer mechanisms in the heterogeneous catalytic reactions. (**A**). direct electron transfer mechanism, (**B**). Sensitizer-assisted electron transfer mechanism and (**C**). Photocatalyst-assisted electron transfer mechanism.

Considering the relative energies at the semiconductor/electrolyte interfaces, there are different mechanisms for electron transfer in the semiconductor catalysts-based chemical reactions, including direct injection, injection through a photosensitizer, and photo-excitation of the utilized catalyst [1]. Figure 1 shows a conceptual illustration of the three mechanisms.

Briefly, the electrons can be directly injected as shown in Figure 1A. In the second mechanism (Figure 1B), a photosensitizer can be used in the photolytic reactions when the electrons cannot be injected from the HOMO of the donor to the conduction band of the catalyst. In photocatalysis, the semiconductor is excited and can deliver and receive electrons to the acceptor and from the donor, respectively, as shown in Figure 1. Previous experimental studies concluded that electron transfer between semiconductors and aqueous redox species occurs exclusively at the semiconductor/electrolyte interface, where two orbitals, one belonging to the semiconductor and the other belonging to the aqueous species, have a similar energy [2].

In the third mechanism, the difference between the energy levels (i.e., conduction band (CB) and the LUMO of the acceptor and the HOMO of the donor and the valence band (VB)) should not be high. For instance, although sphalerite (ZnS) has a high conduction band (−3.46 eV vs. absolute vacuum scale (AVS), −1.04 eV vs. NHE), it was found that this catalyst cannot catalyze hydrogen production a photochemical reaction from water splitting due to the large gap [3]. In other words, a large energy difference results in a slow electron transfer. This phenomenon has been termed as the “*inverted region effect*” [4]. The VB in the oxides semiconductors is primarily corresponding to the O2p orbital. Therefore, their VBs energy levels are near the absolute oxygen electronegativity (–7.54 eV vs. AVS) and much below water oxidation potential (redox potential of O_2_/H_2_O). Consequently, oxygen formation reactions (Equations (3) and (4)) are unlikely to proceed. Therefore, to complete the process, the oxygen generation half-reactions are substituted by utilizing chemical scavengers as electron donors such as sodium sulfide (Na_2_S) [5], sodium sulfite (Na_2_SO_3_) [6], sodium sulfide/sodium sulfite mixture [7], methanol [8], ethanol [9], isopropanol [10], ethylene glycol [11], glycerol [12], glucose [13], lactic acid [14], and triethanolamine [15,16].

To enhance the usability under visible light radiation as well as shorten the gap between OH^−^ or H_2_O reduction (Equations (3) and (4)) and the VB edge, the VB level of these semiconductors has to be shifted upward without changing the CB state. They use oxynitrides or sulfides to take advantage of N2p, and S3p states that lie at more negative potentials than O2p states were exploited as a proposed approach [17,18]. However, these two classes of semiconductors suffer from corrosion due to the fact that if the semiconductor’s standard potential for anodic decomposition is higher in energy than the VB edge, the formed holes can cause oxidation of the semiconductor itself. The Z-scheme photocatalytic system is another approach to overcome the problem mentioned above, as it depends on using two semiconductors with an electron mediator between them [19,20,21,22,23,24]. Due to the accommodation of charge carriers in their respective high-energy states, the Z-scheme requires a substantial overpotential for the total reaction. Another abuse of this system is that it takes eight photons to complete the water-splitting cycle [25]. Moreover, besides the complexity of this system, the reported efficiencies are not as high as expected [26].

To the best of our knowledge, all of the reported photocatalysts for water splitting focus on the excitation of the introduced catalyst(s). However, if a suitable photosensitizer could be invoked (i.e., establishing the second mechanism; Figure 1B), the issues mentioned above might be overcome.

Methylene blue (MB) dye could be successfully exploited as a photosensitizer in the dye-sensitized solar cells [27]. Titanium oxide (TiO_2_) is the oldest and most popular used semiconductor as a photocatalyst for water splitting. Besides its high chemical stability, low cost, and ease of preparation, due to the quantum size effect, colloidal titanium oxide particles have a relatively higher conduction band value than the H_2_O and H^+^ reduction, which is thermodynamically required for hydrogen generation operation. Moreover, the fast electron/hole recombination hurdle can be addressed by the incorporation of a proper dopant which creates a series of intermediate energy levels in the semiconductor band gap [28]. Furthermore, the 1D nanostructure distinctly enhances the electron transfer process [29].

In this study, for the first time, based on our best knowledge, hydrogen production from water splitting was performed based on a photosensitizer-based electron transfer mechanism. Typically, water splitting was performed using SiO_2_ NPs-incorporated TiO_2_ nanofibers as a catalyst and MB dye as a visible light photosensitizer. The 1D nanostructure (nanofiber) composite catalyst was prepared by a simple, low cost, and high yield process (i.e., electrospinning). The results supported the hypothesis that invoking the MB dye as a photosensitizer as the hydrogen was produced under visible light radiation without any significant change in the dye concentration, while under UV the dye almost degraded completely in a short time. Although the hydrogen production rate is high, the main target of this study is opening a new avenue for exploiting photosensitizers in green hydrogen production technology.

## 2. Materials and Methods

### 2.1. Materials

Amorphous silica nanoparticles (SiO_2_ NPs) were extracted by acidic hydrothermal treatment of rice husk according to our previous study [30]. Titanium (1V) isopropoxide (Ti(Iso), 98.0 assay) and N, N-dimethylformamide (DMF, 99.5 assay) were purchased from Junsei Co., Ltd., Tokyo, Japan. Poly(vinyl acetate) (PVAc, MW = 500,000 g/mol) and methylene blue (MB, C_16_H_18_ClN_3_SCl) were obtained from Aldrich, Burlington, MA, USA.

### 2.2. Catalyst Preparation

**Figure 2:**
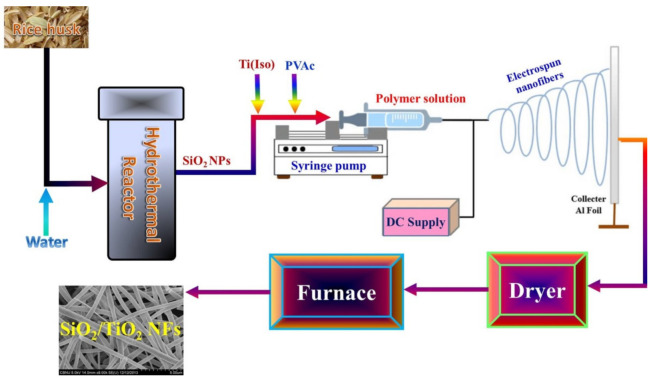
Schematic diagram for SiO_2_ NPs–incorporated TiO_2_ nanofibers synthesis process.

The electrospinning process was utilized to prepare the SiO_2_ NPs-incorporated TiO_2_ nanofibers. Typically, a sol–gel was prepared by mixing titanium isopropoxide (Ti(Iso)), and poly(vinyl acetate) (PVAc, 14 wt% in DMF) with a weight ratio of 2:3, respectively, and then few drops of glacial acetic acid were added until the solution became transparent. The mixing process was carried out at 25 °C using a magnetic stirrer rotating at 150 rpm. To prepare sol-gels containing silica nanoparticles, SiO_2_ NPs were added to the Ti(Iso)/PVAc solution to get final solutions containing 4, and 6 wt% SiO_2_ with respect to the expected produced TiO_2_. Afterward, the solution was stirred under vigorous conditions for 30 min at 25 °C. Later on, a high voltage power supply (CPS-60K02V1, Chungpa EMT Co., Seoul, Korea) was used as a source of the electric field. The sol-gel was supplied through a plastic syringe attached to a capillary tip. A copper wire originating from the positive electrode (anode) connected with a graphite pin was inserted into the sol-gel, and the negative electrode (cathode) was attached to a metallic collector covered by a polyethylene sheet. The solution was electrospun at 16 kV and 15 cm working distance (the distance between the needle tip and the collector). The electrospinning process was conducted at 25 °C in a 40% relative humidity atmosphere. The formed nanofiber mats were initially dried for 24 h at 80 °C under vacuum and then calcined in an air atmosphere at 600 °C for one h at a heating rate of 5 °C/min. Figure 2 represents a schematic diagram for the proposed catalyst synthesis.

### 2.3. Dye Degradation Experiments

Photocatalytic dye degradation experiments have been performed in a glass cylinder batch photoreactor with a volume of 2000 mL. A volume of 500 mL of an aqueous solution of MB (10 ppm concentration) has been prepared. The catalyst has been added to the solution by a weight of 0.05 g. The mixture has been transferred to the reaction vessel (quartz tube) which is exposed to a UV light source by a peristaltic pump. An electrical, magnetic stirrer with a magnetic bar has been used continuously so that the catalyst can be uniformly dispersed in the solution. Figure S1 in the Supplementary Materials displays a schematic diagram for the used setup. The reaction temperature has been maintained at room temperature (27 ± 3 °C) for all of the experimental trials. Samples have been collected using 5 mL plastic test tubes. The concentration of the dye has been determined using a spectrophotometer instrument by checking the absorbance at λ = 664 nm.

### 2.4. Hydrogen Production

Hydrogen production experiments have been performed in Pyrex spherical glass with a volume of 500 mL. 0.05 g of the prepared catalyst has been added to a 100 mL of methylene blue dye aqueous solution (10 ppm concentration). The plug has been closed and connected with a tube in a conical flask, the measuring cylinder filled with water, and upside downs in a glass beaker. The mixture has been exposed to visible light (Halogen lamp, 2000 watt, ORSAM Entertainment and Industry, Mexico City, Mexico). An electrical, magnetic stirrer with a magnetic bar has been used continuously to keep the catalyst uniformly dispersed in the solution. The experiments have been repeated three times, and the introduced data are the average of the experimental results.

### 2.5. Characterization

A field-emission scanning electron microscope equipped with an EDX analysis tool (FESEM, Hitachi S-7400, Tokyo, Japan) was used to investigate the surface morphology. However, the phase and crystallinity were detected by Rigaku X-ray diffractometer (XRD, Rigaku, Tokyo, Japan) with Cu Kα (λ = 1.540 Å) radiation over Bragg angle ranging from 10 to 80°. Standard and high-resolution images were described by transmission electron microscope (TEM, JEOL JEM-2010, Tokyo, Japan) operated at 200 kV. The concentration of the dye in the photodegradation was investigated by spectroscopic analysis using an HP8453UV–visible spectroscopy system (Conquer Scientific, Poway, CA, USA). Furthermore, the spectra obtained were analyzed by the H.P. Chemi Station software 5890 Series (Agilent Technologies, Santa Clara, CA, USA). The obtained spectra were utilized to estimate the bandgap energies using Tauc modified equation. Tauc proposed utilizing optical absorption spectra to assess the bandgap energy of amorphous semiconductors [31]. Davis and Mott explored his concept further [32]. The Tauc technique is predicated on the supposition that the energy-dependent absorption coefficient (*α*) can be represented using the equation below [33].

(5)  α·hv1γ=Bhv−Eg

In this equation, the symbols *h*, *v*, and *E_g_* represent the Planck constant, the photon’s frequency, and the bandgap energy, respectively, and *B* is a constant. The *γ* factor is related to the nature of the electron transition; its value equals 0.5 and 2 for the direct and indirect transition band gaps, respectively. Usually, the bandgap energy is estimated from the diffuse reflectance spectra. However, Kubelka–Munk function can be applied to transfer the reflectance spectra to the corresponding absorption spectra as follow:

(6) $$F\left(R_{\infty }\right)=\frac{K}{S}=\frac{{\left(1-R_{\infty }\right)}^{2}}{2R_{\infty }}$$

where $R_{\infty }=R_{sample}/R_{standard}$ is the reflectance of an infinitely thick specimen, while *S* and *K* are the scattering and absorption coefficients, respectively. Updating Equation (5) by replacing *α* by *F*(*R*_∞_) results in having this new equation form:

(7) FR∞·hv1γ=Bhv−Eg

Gas detection was done by Shimadzu’s GC-2025 (Shimadzu, Kyoto, Japan) capillary gas chromatograph in a batch mode. Typically, the gas was injected into the instrument using a special syringe.

## 3. Results

### 3.1. Criteria of the Required Catalyst

Interestingly, the CB edge of titanium oxide (−4.21 eV vs. AVS and −0.29 eV vs. NHE) is close to the reduction potential of water (redox potential of H_2_/H^+^), which encourages utilization in water photo splitting operation. Moreover, the nanoparticles’ aqueous solution suspension possesses a further advantage for water reduction due to the quantum effect. However, the very low VB, compared to the oxidation potential of water (O_2_/H_2_O couple), is a highly undesired characteristic due to the severe difficulty of performing oxygen generation reaction(s) directly over the surface of TiO_2_ nanoparticles. Moreover, the low VB energy edge enlarges the bandgap, requiring UV irradiation for photo-excitation. Figure S2 in the Supplementary Materials displays the CB and VB states for TiO_2_ compared to other semiconductors.

Fast electron/hole recombination is another obvious problem facing most of semiconductors. One successful solution for addressing this issue is doping the semiconductor with proper impurities. Impurities incorporation can result in establishing electron acceptor (and/or donor) state levels within the semiconductor band gad edges, which affects the electron/hole recombination rate [1]. Metal ions doping restricts the electron-hole recombination and enhances the quantum efficiency due to changes in the crystal lattice structure (and/or crystal parameters) of the host semiconductor [34]. Metallic nanoparticle incorporation can solve this problem by forming the Schottky barrier due to the metal-semiconductor junction. However, not all metal-semiconductor junctions create a rectifying Schottky barrier. An ohmic contact is a metal–semiconductor junction that conducts current in both directions without rectification, possibly due to a low Schottky barrier [35]. The presence of the amorphous phase within the crystals of the semiconductor can suppress the electron/hole recombination due to the formation of different pathways for the electrons [36]. However, insertion of an amorphous dopant can increase the required energy for the photo-excitation. In other words, it shifts the band gap energy to be within the UV region. Nanofibers own their distinguished characteristics compared to the other nanostructures due to the large axial ratio. For semiconductors, the nanofibrous morphology distinctly facilitates the electron transfer process [37].

The aforementioned explanation targeted the preparation of TiO_2_-based nanofibers containing amorphous dopants to ensure trivial photo-excitation under visible light radiation and low electron transfer resistance.

### 3.2. Catalyst Characterization

#### 3.2.1. Structure and Morphology

Electrospinning is the most utilized process for polymeric and inorganic nanofibers fabrication due to its simplicity, high yield, low cost, and ease of controlling for the morphology of the final product [38]. However, due to the necessary calcination step, in the case of inorganic nanofibers production, to remove the used polymer, using a precursor having a high polycondensation tendency is mandatory [39]. It is known that alkoxides possess a high propensity for polycondensation. Consequently, Ti(IsO) (the used precursor in this study) was widely used to prepare TiO_2_ nanofibers [40].

**Figure 3:**
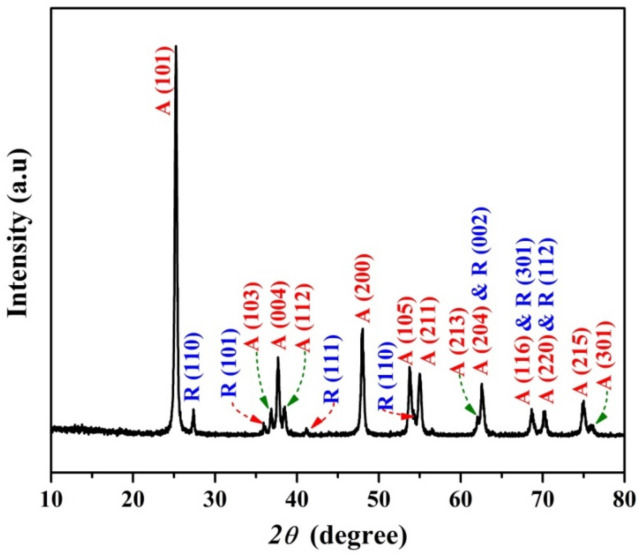
XRD pattern for the produced SiO_2_ NPs-incorporated TiO_2_ nanofibers.

Figure 3 displays the XRD diffraction patterns of the as-obtained powder after the calcination process. As shown, anatase TiO_2_ is the predominant phase in the produced powder. Typically, the firm diffraction peaks at 2*θ* values of 25.15°, 36.95°, 37.64°, 38.55°, 48.05°, 53.85°, 55.07°, 62.20°, 62.67°, 75.30°, and 76.00° corresponding to the crystal planes (101), (103), (004), (112), (200), (105), (211), (213), (204), (215), and (301), respectively, confirm the formation of anatase titanium dioxide [JCPDS card no 21-1272]. With small content, rutile phase has also been having detected in the obtained pattern as it could be concluded from the observed peaks at 27.45°, 36.09°, 41.23°, 54.32°, 62.72°, 69.00°, and 70.69° corresponding to the crystal planes of (110), (101), (111), (211), (002), (301), and (112), respectively [JCPDS card no 21-1276]. However, no peaks could be detected representing the added silica since the used dopant has an amorphous structure that was approved in our previous study about extracting silica nanoparticles from the rice husk [30].

X-ray diffraction analysis is based on the reflection of the X-rays on the highly occupied atomic plans in the crystals of the investigated sample, so it is not an appropriate analytical tool for amorphous materials. The added amorphous silica nanoparticles could be detected in the prepared nanofibers by analyzing the Energy-dispersive X-ray spectroscopy (EDX). EDX is an analytical technique for determining a sample’s elemental composition or chemical characterization. It is based on an interaction between an X-ray source and a sample. Its characterization powers are mainly attributable to the fundamental concept that each element has a unique atomic structure, resulting in a distinct collection of peaks on its electromagnetic emission spectrum.

**Figure 4:**
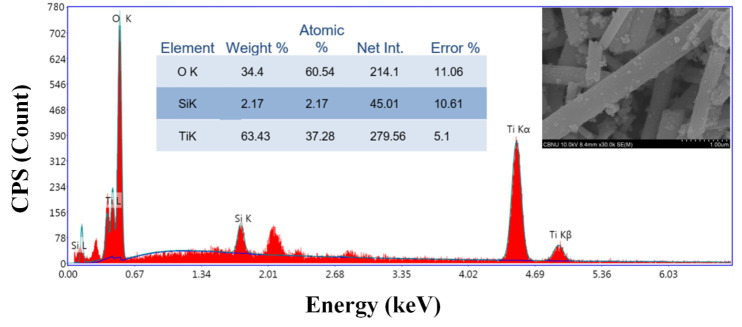
EDX analysis for the produced SiO_2_ NPs–incorporated TiO_2_ nanofibers. The inset table displays the elemental analysis data, and the image represents the FE SEM result.

Figure 4 shows the EDX spectrum for the prepared nanofibers. As shown, the peaks corresponding to silicon, titanium, and oxygen strongly appeared, which concludes that the investigated sample is composed of these three elements and consequently confirms incorporation of the SiO_2_ NPs in the produced nanofibers. The inset image presents the FE SEM results. It can be concluded that the calcination of the Ti(Iso)/SiO_2_ NPs/PVAc nanofiber mats did not wholly destroy the nanofibrous morphology, as broken and long nanofibers were obtained (as can be seen in the image). It is worth mentioning that beads-free and smooth nanofibrous morphology were the main characteristics of the original electrospun nanofibers regardless of the SiO_2_ NPs content (data are not shown).

Worth mentioning, undetectability of SiO_2_ in the XRD results was the desired conclusion since it denotes that the achieved calcination process did not alter the amorphous structure of the used dopant to be crystalline, so the final composition is TiO_2_-based nanofibers having a larger bandgap energy compared to the pristine TiO_2_ nanofibers.

#### 3.2.2. Internal Structure

**Figure 5:**
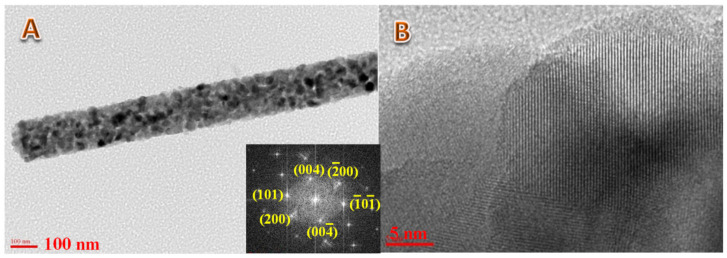
Normal; (**A**) and high resolution; (**B**) TEM images for the produced nanofibers. The inset represents the SAED result.

Figure 5 displays an internal structure investigation using the transmission electron microscope analyses (TEM). As it can be concluded from the results in the selected area electron diffraction (SAED) image, the inset in Figure 5A, the TiO_2_ matrix in the prepared nanofibers is highly crystalline. The main crystal plans are displayed as clear spots in the SAED representative matrix (Ewald sphere construction). As shown in the inset in Figure 5A, the main crystal plans clearly appear and are indexed on the SAED network. Moreover, the high-resolution TEM image (Figure 5B) further affirms the high crystallinity of the nanofiber matrix. As shown, the crystal planes appear in clear parallel lines (the red dot circles). Moreover, the obtained interplanar distance matches the standard value of the TiO_2_ crystal.

#### 3.2.3. Optical Properties

**Figure 6:**
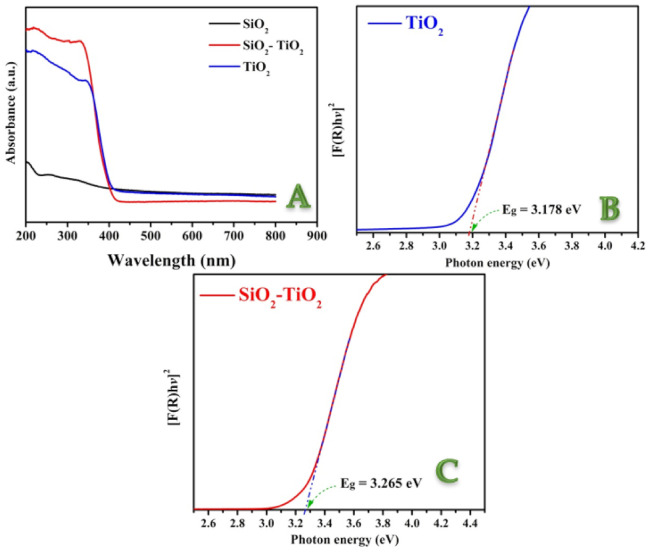
UV-vis. spectra for SiO_2_ NPs, and pristine and SiO_2_–incorporated TiO_2_ nanofibers (**A**); and Tauc plots for determination the bandgap energies for the pristine and SiO_2_–incorporated TiO_2_ nanofibers ((**B**) and (**C**), respectively).

To predict photophysical and photochemical characteristics of semiconductors, a precise estimate of the bandgap energy is required. This parameter is frequently used while discussing photocatalytic characteristics of semiconductors. Figure 6A shows the UV-vis spectra for the used SiO_2_ NPs and pristine and SiO_2_–incorporated TiO_2_ nanofibers. Figure 6B,C display the reflectance spectra transformed according to Equation (7) plotted against the photon energy for the pristine and SiO_2_-incorporated TiO_2_ nanofibers, respectively. The semiconductor can be characterized by the region showing a steep that reflects the linear increase of light absorption upon increasing the incident light energy. An estimated value for the bandgap energy can be determined from the *x*–axis intersection point of the linear fit of the Tauc plot. As shown in the figures, SiO_2_ NPs increased the bandgap energy. Numerically, the band energies for the pristine and modified TiO_2_ nanofibers are 3.187 and 3.265 eV, respectively. Therefore, it can be claimed that the proposed incorporation resulted in shifting the required photons for excitation to be within the UV zone and farther from the visible light region.

### 3.3. Catalyst Activity

#### 3.3.1. Dye Photo Degradation

**Figure 7:**
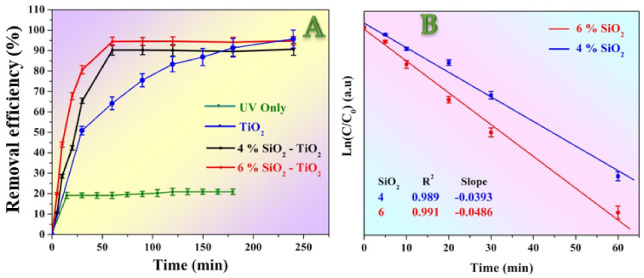
Natural methylene blue dye removal rate (UV only) and in the presence of the prepared nanofibers photocatalysts under U.V. irradiation; (**A**), and linear plot of Ln(C/C_0_) vs. time for the photocatalytic degradation results using SiO_2_–incorporated TiO_2_ nanofibers; (**B**).

Degradation of methylene blue is widely used as a model to investigate the efficacy of the photocatalysts toward wastewater treatment process [41]. In this regard, many materials were introduced, including magnetite nanoparticles and the corresponding composites [42,43,44]. In this study, dye degradation results under UV irradiation are demonstrated in Figure 7. It was discovered that the methylene blue dye has a little self-cracking tendency under the UV light; almost 20% of the dye decomposes after 15 min. Later on, no considerable change in the concentration was noticed, as shown in Figure 7A. However, the presence of the catalyst has a distinct impact on the degradation rate, as shown in the figure. Whereby the composition of the catalyst affects the photo degradation operation. Addition of the amorphous silica NPs results in performing the maximum removal efficacy quickly compared to SiO_2_–free TiO_2_ nanofibers. Typically, incorporation of the amorphous NPs led to getting the maximum efficiency after 60 min, which is one-third for the required time in the case of pristine TiO_2_ nanofibers; 180 min. Moreover, more than 90% of the dye has been decomposed within this relatively short time. This finding can be attributed to the successive role of the used SiO_2_ NPs in retarding the electron/hole recombination process. It is worth mentioning that increasing the silica content from 4 to 6 wt% did not strongly affect the dye degradation operation as, after 60 min, 90.3, and 94.5% removal has been achieved by utilizing the 4 and 6 wt% silica content, respectively.

It was realized that the kinetics of the dye degradation using the SiO_2_–incorporated TiO_2_ nanofibers resembles the first-order reactions. To prove this hypothesis, the application of the first reaction kinetic model on the obtained data has been investigated. As shown in Figure 7B, linear fitting can highly match the experimental data. Typically, the relation between Ln(C/C_0_) versus time could be linearly fitted with very acceptable linear regression factors (*R*^2^; 0.989 and 0.991 for the 4 and 6 wt% silica, respectively). However, the first-order reaction kinetic model could not be applied successfully on the dye degradation results using the pristine TiO_2_ nanofibers as the regression factor was a little far from the unity; data are not shown. It is worth mentioning that silica NPs have an adsorption capacity [45,46]. Therefore, the enhancement in the photocatalytic activity of the introduced photocatalyst can be partially attributed to the adsorption capacity since the used NPs are mostly incorporated inside the TiO_2_ nanofiber matrix, as shown in the TEM image. However, the adsorption effect cannot be neglected.

#### 3.3.2. Water Photo Splitting

**Figure 8:**
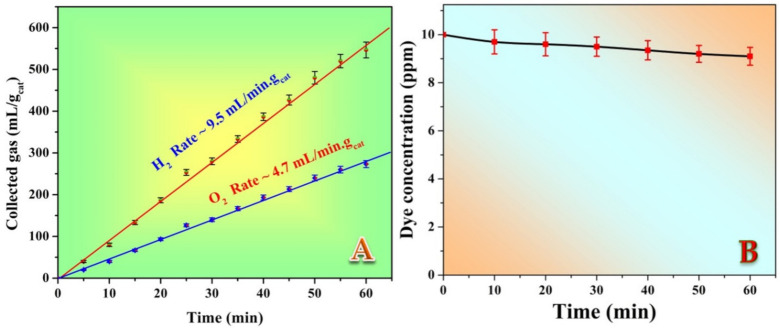
Hydrogen and oxygen production rates under visible light irradiation using SiO_2_–incorporated TiO_2_ nanofibers (6 wt% sample) and methylene blue as photosensitizer; (**A**), and dye photodegradation rate during the water splitting process; (**B**).

On the other hand, methylene blue could successfully enhance the water photo splitting reactions under visible light radiation, as shown in Figure 8. As shown in Figure 8A, hydrogen and oxygen were obtained with a very acceptable production rate compared to many reported photocatalysts [26,47,48,49,50]. Moreover, the obtained hydrogen and oxygen rates follow the stoichiometry of water splitting reaction (~4.02 × 10^−4^ and 1.96 × 10^−4^ mol/min/g_cat_, respectively). Consequently, it can be claimed that water splitting process was performed since no other gases were detected. Interestingly, as it could be seen in Figure 8B, the concentration of the utilized MB dye decreased very little throughout the experiment (from 10 to 9.1 ppm), which could be attributed to the self-decomposition of the dye under visible light. Moreover, the hydrogen/oxygen production rate ratio almost matches the stoichiometry of the water-splitting reaction. This finding is very important as it confirms exploiting of the methylene blue dye as a photosensitizer during the water photo splitting process. It is worth mentioning that for the prepared formulations, SiO_2_ NPs content did not considerably affect the catalyst performance; the introduced data belonged to the 6 wt% samples.

### 3.4. Mechanism Study

**Figure 9:**
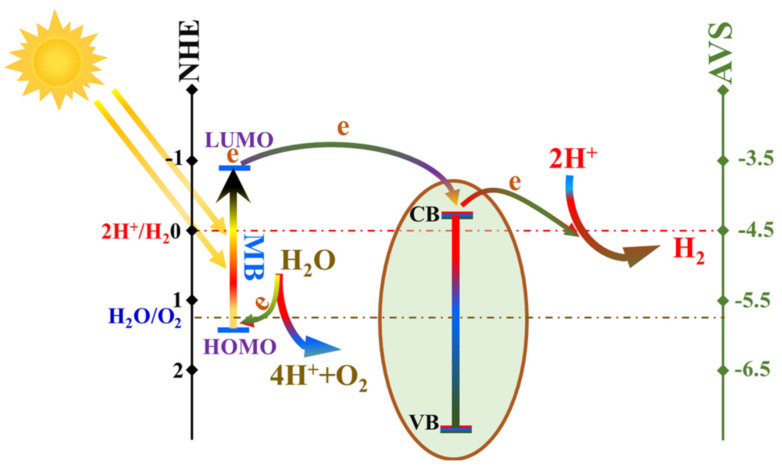
Conceptual illustration for the mechanism of water splitting under visible light radiation using methylene blue dye as a photosensitizer.

To understand the mechanism of water splitting using methylene blue as a photosensitizer, Figure 9 was drawn as a conceptual illustration. Theoretically, the bandgap and conduction band energies for TiO_2_ are 3.2 and −0.29 eV vs. NHE (3.2 and −4.21 eV vs. AVS), respectively. Therefore, the proposed modification process did not considerably change the energy states of the introduced composite nanofibers compared to the theoretical values as the estimated bandgap energy is 3.265 eV. Considering that incorporation of impurities usually affects the valance band states, it can be claimed that the conduction band for the prepared nanofibers is close to the theoretical value (−0.29 eV vs. NHE). Thus, the valance band is around 2.975 eV. Consequently, the conduction and valance bands energies for the introduced catalyst could be graphically displayed in Figure 8. In the literature, the LUMO and HOMO of methylene blue dye could be estimated using the DFT B3LYP/16301G* method as −0.88 and 1.55 eV, respectively [51]. Consequently, the bandgap is determined as 2.43 eV. Accordingly, this visible light photon energy is adequate for the excitation of the electrons in the HOMO of this dye to jump to the LUMO orbitals. Thus, it could be exploited as a photosensitizer in the DSSCs [27]. Based on the energy states of the present system and simulating to DSSC, the excited electrons in the LUMO of the methylene blue dye can transfer to the conduction band of the used catalyst. Then, they are pumped to the hydrogen ions to achieve the reduction reaction in the water-splitting process. On the other hand, the energy state of the formed holes in the HOMO of the dye is adequate to receive the electrons from the water oxidation reaction. In other words, the potential difference between the water oxidation reaction (1.22 eV) and the HOMO of the dye (1.55 eV), 0.33 eV, is convenient for electron transfer to fill the empty holes and consequently complete the water-splitting process.

In several studies, it was reported that the main species behind the organic dyes photodegradation is the hydroxide radical (OH^●^) and/or hydrogen peroxide (H_2_O_2_) [51]. Desirably, the reaction potentials of these active species are adequate with the energy states of the prepared catalyst as follow:

O_2_ + 2H_2_O + 2e^−^ → H_2_O_2_ + 2OH^−^   *E*_0_ = −0.15 V (8)

2H_2_O → 2OH^●^ + 2H^+^ + 2e^−^   *E*_0_ = 2.85 V (9)

**Figure 10:**
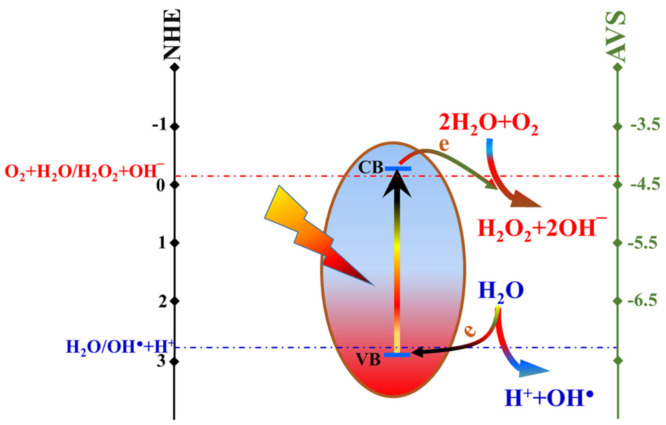
Conceptual illustration for the mechanism of methylene blue dye photo degradation under UV light irradiation using the prepared SiO_2_–incoportaed TiO_2_ nanofibers as photocatalyst.

Consequently, as shown in the conceptual illustration (Figure 10), upon exposure to the UV irradiation, hydrogen peroxide can be easily formed due to the easy transfer of the excited electron from the prepared semiconductor conduction band proceeding the reduction reaction; Equation (8). Similarly, due to the very short energy gap between the potential of hydroxide radical formation reaction (Equation (9)) and the valance band of the excited photocatalyst (2.975 eV), the oxidation reaction can be carried out and deliver the electrons to holes in the valance orbitals. This proposal explaining the methylene blue photodegradation process mechanism is highly accepted since almost all of the reports discussing the photodegradation of the organic pollutants using TiO_2_-based photocatalysts are based on Equations (8) and (9).

## 4. Conclusions

Silica NPs-incorporated titanium oxide nanofibers can be prepared by calcination of electrospun nanofiber mats composed of silica NPsN.P.s, titanium isopropoxide, and poly(vinyl acetate) under air atmosphere. Incorporation of the amorphous silica nanoparticles leads to little enlargement of the bandgap energy (3.265 eV) of the produced TiO_2_-based nanofibers that makes this photocatalyst applicable under the UV irradiation. Methylene blue dye has a convenient band gap energy to photo excite under visible light radiation; consequently, it can be used as a photosensitizer in the water photo splitting process using SiO_2_-TiO_2_ nanofiber catalyst with a relatively good hydrogen production rate (4.02 × 10^−4^ mol/min/g_cat_). At the same time, the role of the used semiconductor composite is receiving the excited electron from the dye LUMO energy state and delivering it to the hydrogen ions reduction reaction. On the other hand, under UV light irradiation, a relatively complete (93% removal efficiency) degradation of the methylene blue dye can be achieved due to the photo-excitation of the proposed mixed oxides semiconductor. Overall, a proper organic substance can be invoked as a photosensitizer in the presence of a large bandgap energy semiconductor to produce hydrogen from water splitting effectively.

## Data Availability

The data presented in this study are available upon request from the corresponding author.

## Supplementary Materials

The following supporting information can be downloaded at: https://www.mdpi.com/article/10.3390/polym14030523/s1, A schematic diagram for the dye degradation setup and a graph for the bandgap energies of the popular photocatalysts are available online. Figure S1 displays a schematic diagram for the used dye degradation setup, Figure S2 shows conduction and valance bands position for TiO_2_ and other semiconductors.
