# Supplementary material for: Methylene Blue Dye as Photosensitizer for Scavenger-Less Water Photo Splitting: New Insight in Green Hydrogen Technology
Source: Polymers (Basel). 2022 Jan 27;14(3):523. doi: 10.3390/polym14030523 (PMC8839752; doi:10.3390/polym14030523)
Supplement: Supplementary file 1 [file polymers-14-00523-s001.zip › polymers-1511655-supplementary.pdf]

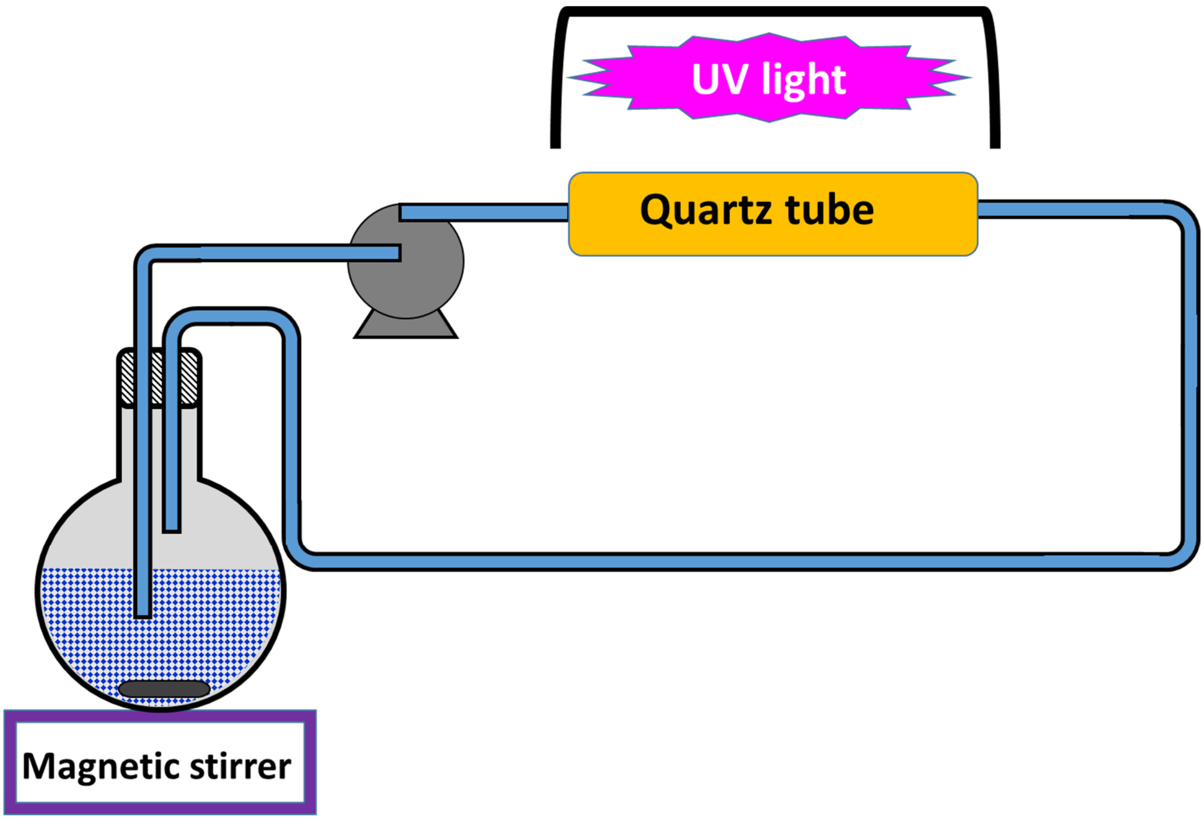

Figure S1. Schematic diagram for the used dye degradation setup.

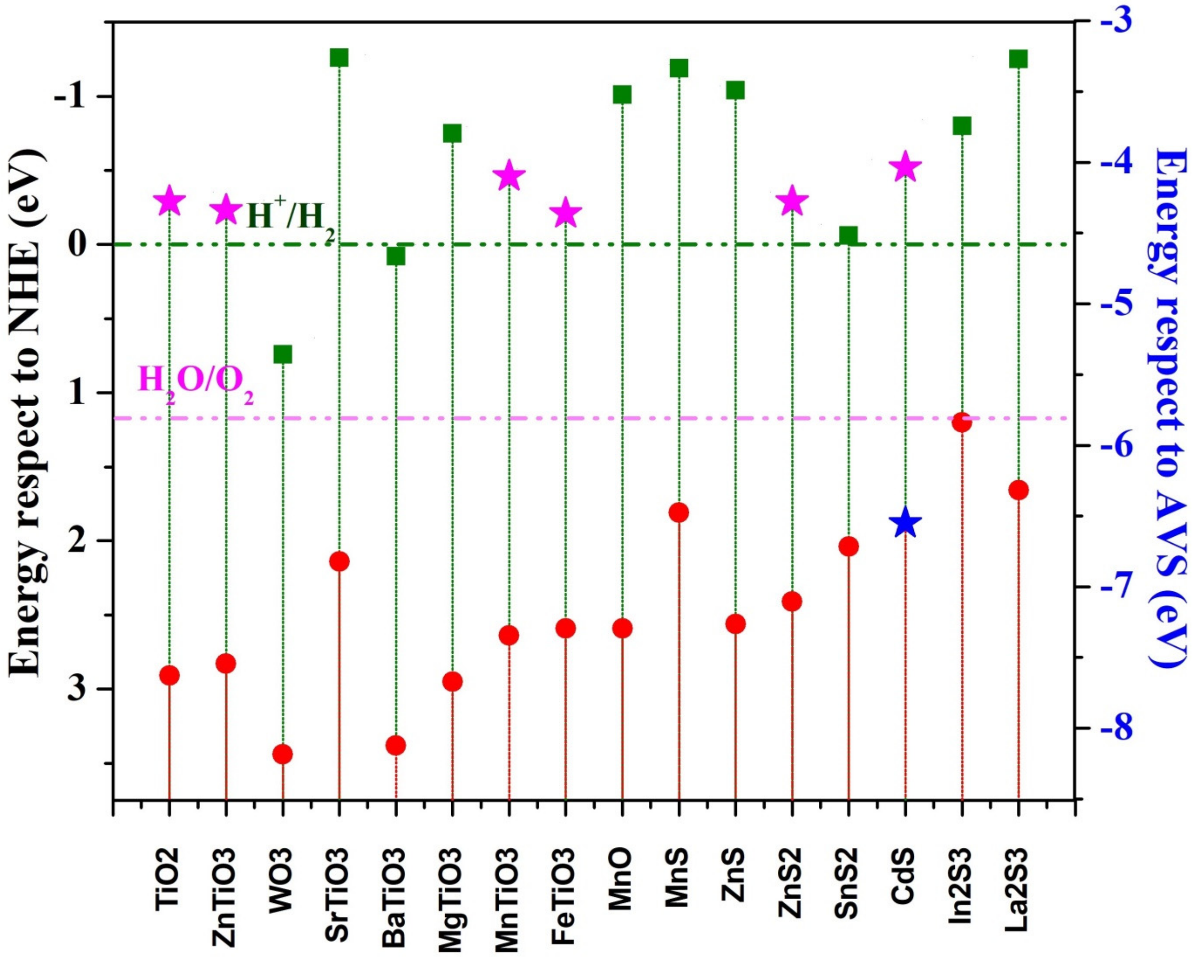

Figure S2. Conduction and valance bands position for TiO_2 and other semiconductors.
